# Mechanism Analysis of Rock Failure Process under High-Voltage Electropulse: Analytical Solution and Simulation

**DOI:** 10.3390/ma15062188

**Published:** 2022-03-16

**Authors:** Pingping Rao, Peihao Ouyang, Sanjay Nimbalkar, Qingsheng Chen, Jifei Cui, Zhilin Wu

**Affiliations:** 1 Department of Civil Engineering, University of Shanghai for Science and Technology, 516 Jungong Road, Shanghai 200092, China; 203642074@st.usst.edu.cn (P.O.); cuijifei@usst.edu.cn (J.C.); wuzhilin0606@163.com (Z.W.); 2 School of Civil and Environmental Engineering, University of Technology Sydney, 15 Broadway, Ultimo, Sydney, NSW 2007, Australia; sanjay.nimbalkar@uts.edu.au; 3 Technology Research Center of Ecological Road Engineering, Hubei University of Technology, Wuhan 430068, China; chqsh2006@163.com

**Keywords:** high-voltage pulsed fragmentation, equivalent circuit, shock wave, crack development, analytical solution and simulation, rock failure process analysis

## Abstract

This work aims to investigate and analyse the mechanism of rock failure under high-voltage electropulses in order to evaluate and increase the efficiency of high-voltage pulse technology in geological well drilling, tunnel boring, and other geotechnical engineering applications. To this end, this paper discusses the equivalent circuit of electric pulse rock breaking, the model of shock wave in electro channel plasma, and, particularly, the model of rock failure in order to disclose the rock failure process when exposed to high-voltage electropulse. This article uses granite as an example to present an analytical approach for predicting the mechanical behaviour of high-voltage electropulses and to analyse the damage that occurs. A numerical model based on equivalent circuit, shock wave model, and elasto-brittle failure criterion is developed for granite under electropulse to further examine the granite failure process. Under the conditions described in this study, and using granite as an example, the granite is impacted by a discharge device (Marx generator) with an initial voltage *U*_0_ that is 10 kV and a capacitance *F* that is 5 µF before it begins to degrade at about 40 µs after discharge, with the current reaching its peak at approximately 50 µs. The shock wave pressure then attains a peak at about 70 µs. Dense short cracks form around granite and the dominant cracks grow to an average length of about 20 cm at around 200 µs. The crack width *d_cr_* is predicted to be approximately 1.6 mm. This study detects dense cracks in a few centimetres surrounding the borehole, while around seven dominant cracks expand outward. The distribution of the length of the dominating cracks can be inhomogeneous because of the spatial heterogeneity of granite’s tensile strength, however the heterogeneity has an insignificant effect on the crack growth rate, total cracked area, or the number of main cracks. The mechanism of rock failure under electropulse can be well supported by the findings of numerical simulations and analytical studies.

## 1. Introduction

With the growth of geotechnical underground techniques such as geological well drilling and tunnel boring, traditional mechanical methods for rock breaking have been constrained (Yan et al., 2012) [1]. These old traditional mechanical methods, such as mechanical drilling, the drilling–blasting method and full-face tunnel boring machine (TBM), all have limitations, including low drilling efficiency, high drilling costs, and stricter geological criteria (Wang et al., 2017) [2]. Additionally, the controllability of the aforementioned standard rock breaking technologies is constrained by the complicated geological conditions encountered during deep mining and ultra-deep mining at elevated temperatures and pressures (Zhu et al., 2021a) [3]. Thus, it is critical to investigate a method for rock-breaking drilling that is highly efficient, controllable and widely applicable.

High-voltage discharge rock breaking is a highly effective technique that has been widely used in the fields of underground drilling, mineral processing, micro-ore decomposition, and incrustation cleaning (Burkin et al., 2009a; Yan et al., 2016; Wang et al., 2015; Duan et al., 2015; Touzé et al., 2016; Yudin et al., 2019) [4,5,6,7,8,9]. The advantage of high-voltage discharge drilling technology, which is close to industrialization at the moment (Yudin et al., 2019; Dzhantimirov et al., 2005; Park et al., 2013) [9,10,11], is its high breaking efficiency, controllability and low cost in deep welling (Biela et al., 2009) [12].

Several scholars have explored the electropulse drilling technology and its rock-breaking mechanism over the past several decades. There are some outcomes from the study of electrical pulse energy optimization and rock-breaking efficiency. Andres (1989) [13] has investigated the influence of varying voltage levels and energy densities on the ability of granite to be broken by electropulses and discovered a significant variation in the rock-breaking effect at varying voltage and energy levels. Lisitsyn et al. (1998) [14] conducted electro breaking studies on granite and concluded there to be a greater discrepancy in rock-breaking efficiency at various electrode spacing and voltages. According to Andres et al. (2001) [15], rock breaking is caused by a plasma channel created by an electrical pulse inside the rock which explodes and sends shock wave fragments through it. On this premise, Timoshkin et al. (2004) [16] have argued that the primary goal of energy optimisation for electropulses should be to improve the work performed on the arc channel. Zhang et al. (2012) [17] conducted an experimental study on rock breakdown in deionized water and discovered that porosity has a substantial effect on the electro breakdown field intensity. Burkin et al. (2009b) [18] used simulation to investigate the power characteristics of wave disturbances caused by an electropulse discharge channel in rock, followed by the prediction of a rock failure pattern based on the discharge circuit parameters. Following that, Cho et al. (2016) [19] studied the fragmentation processes in rocks subjected to high-voltage pulse discharges using a high-resolution X-ray CT system and concluded that electropulse-induced rock fractures should originate from dielectric breakdown-induced body forces. Zhu et al. (2021a) [3] investigated the parameters controlling the development of plasma channels in order to uncover a portion of the rock-breaking process and discovered that formation pressure had little effect on rock failure.

Early studies investigated the electropulse-induced damage to rock on a simulated and experimental level. Previous research on high-voltage electro-pulse breaking rock has largely ignored analytical methods of examining the mechanism of rock failure (Zhu et al., 2021a) [3], similarly neglecting the shock wave propagation and rock damage types (Cho et al., 2016; Zhu et al., 2021b) [19,20]. Indeed, the majority of elastic-brittle damage occurs in hard and middle-hard rocks (Li et al., 2015; Yang et al., 2016; Li et al., 2021a) [21,22,23]. When shock waves propagate through rocks, they cause damage in a variety of ways (compressional, shear, and tensile) (Zhu et al., 2008; Wei et al., 2021) [24,25]. Simultaneously, shock waves may subject the rock to a combined tension–compression triaxial stress state (Dai, 2001; Li et al., 2012) [26,27]. Furthermore, under a high-voltage electropulse, the rock is not only subjected to dynamic loading such as shock wave, but also high-temperature plasma and explosive products in the form of quasi-static effects that continue to damage the rock in different time scales (Ma and An, 2008; Vogler et al., 2020; Zuo et al., 2020) [28,29,30]. Meanwhile, the heterogeneity of the rock causes a change in the distribution of stress, which may lead to fractal cracking or secondary cracking (Yudin et al., 2019; Kutter and Fairhurst, 1971) [9,31]. This adds complexity to the examination of the rock collapse process and the formation of damage. Thus, additional research is required on the propagation form and attenuation law of shock waves generated by electrical pulses in rocks, as well as the failure form of rock, with the goal of elucidating the rock failure process caused by electropulses and thus increasing the efficiency of rock breaking.

To analyse the mechanism of rock failure caused by electropulses, this paper examines the mechanism of electropulse digging in rock first. Then, using granite as an example, the damage form and region of granite subjected to an electric pulse are discussed analytically (crushed, fracture, crack zones). Additionally, this study proposes a numerical model for simulating the granite failure process and fracture propagation. Following that, the results are evaluated against experiments and simulations from a variety of sources. Meanwhile, the crack lengths determined using the analytical and simulation approaches described in this paper are shown to be well in agreement. Finally, a simulation of the granite failure process is undertaken, including circuit design, shock wave creation, and propagation, in order to further understand granite damage caused by electropulses.

## 2. Rock Electropulse Broken Process

The results of high-voltage electro-pulse rock breaking experiments show that the damage to surrounding rock exhibits similar damage processes and regional characteristics as conventional coupled blasting. (Cho et al., 2016) [19] In other words, when the rock is detonated, the shock wave first acts on the discharge channel–rock boundary and crushes the rock around the arc borehole. During the process of shock wave propagation, the blast wave attenuates to a stress wave, which imposes tensile damage on the rock surrounding the borehole and forms radial and hoop cracks at a certain distance to the it (Dai, 2001) [26].

The strength of the induced shock wave is far less than the dynamic compressive strength of rock, and the borehole surrounding rock is not subjected to much intensive compressive damage when the shock wave in the electric channel, within the vicinity of the explosive, impacts on the rock surface of the borehole. As a result, hard rock is rarely prone to crushing damage when subjected to an electric pulse.

High-intensity shock waves eventually attenuate to low-strength stress waves as the propagation distance of the shock wave increases (Dai, 2001) [26]. The surrounding rock of the borehole is continuously compressed in the radial direction, resulting in tensile deformation in the tangential direction. The tensile strength of rock is considered to be 0.02 to 0.10 times that of its compressive strength. (Li et al., 2016) [27]. When the surrounding rock’s tangential stress reaches its ultimate tensile strength, it cracks in the tangential direction, forming radial cracks that penetrate the borehole’s crushing or compacting zone. The stress wave in the surrounding rock decreases as nearby radial cracks form, the original compressive de-formation energy accumulated in the cavity borehole surrounding rock releases and generates tensile stress in the opposite direction of the stress wave, causing counter-radial movement of the rock and the formation of a hoop tensile crack in the surrounding rock.

**Figure 1:**
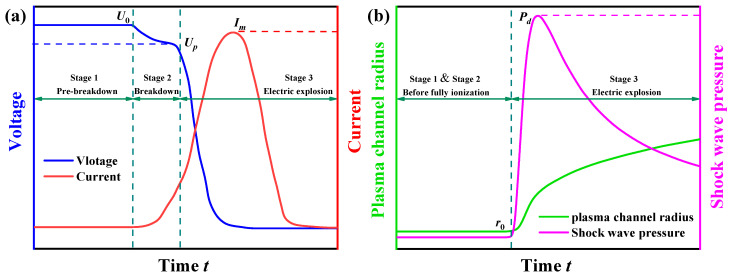
Three stages of the electrical pulse process about (**a**) the voltage and current of plasma channel, and (**b**) shock wave pressure and radius of plasma channel. *U*_0_ is the peak voltage; *U_p_* is the initial voltage of the channel; *I_m_* is the peak current; *r*_0_ is the initial radius of the plasma channel; *P_d_* is the peak shock wave pressure.

In this study, the rock failure process under a high-voltage electropulse is divided into three phases, as shown in Figure 1a. The initial stage is termed pre-breakdown. At this stage, high-voltage pulse electrodes are applied to the rock, but no current is produced or energy dissipated in the rock due to the absence of an analogous loop. Meanwhile, the resistance of the granite near the electrodes rapidly decreases and is broken down first. The breakdown point can be conceived of as an extension of the electrode, and it is at the breakdown point that the next breakdown point is sought. When a breakdown pathway forms within the rock and connects the two electrodes, the rock is electric pierced. Then the second stage occurs, which is the production of a small discharge precursor within the rock, which results in a slight drop in voltage from *U*_0_ to *U_p_*, current in the circuit, and the formation of the initial plasma channel. After the complete formation of the plasma channel, the electric explosion stage (third stage) is entered. The voltage and current are abruptly changed, and the circuit’s energy is completely released until the voltage approaches zero.

The energy *W*_ch_ transported from the circuit to the plasma channel can be split into two components: the mechanical work *W*_ws_ exerted on the surrounding rock as the electric channel expands, and the channel’s internal energy *W*_in_ (Burkin et al., 2009a) [4]. Internal energy is employed to maintain the channel and should cause minimal damage to the surrounding rock. The shock wave can be thought of as the mechanical work done to the surrounding rock. The shock wave’s radius can be equal to the channel’s radius. There will be no shock wave production in the first stage due to the absence of a current loop. The second stage involves the formation of the plasma channel in the discharge circuit; however, because the capacitor voltage consumption is slow, the shock wave cannot be created. In the third stage, a substantial quantity of energy is rapidly released into the plasma channel, causing it to rapidly heat up and expand outward, forming an overpressure shock wave. The evolution of the shock wave pressure and the channel radius over time is depicted in Figure 1b.

**Figure 2:**
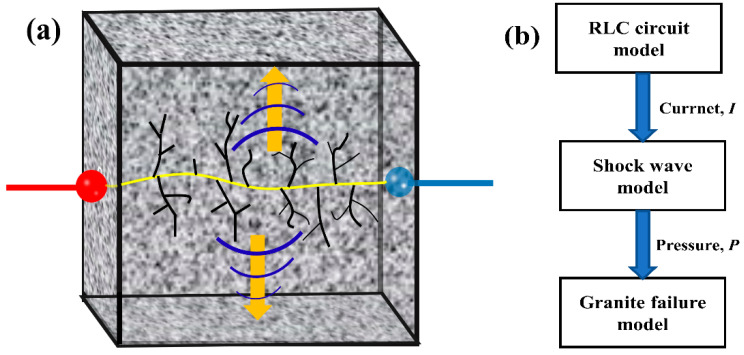
(**a**) Granite broken forms and (**b**) broken process under electropulse.

As the shock wave created by the arc channel and the surrounding rock is directly coupled in this work, it may be assumed that the impedance of the rock medium and the shock wave are perfectly matched, allowing the shock wave pressure to be transmitted completely into the rock. The shock wave delivered disrupts and destroys the rock. According to blasting, damage, and fracture mechanics, and taking into account the rock’s compression–shear–tension triaxial stress state, this paper employs an analytical and simulation approach to calculate the length of the cracks using the RLC (a kind of circuit structure composed of resistance (*R*), inductance (*L*) and capacitance (*C*)) equivalent circuit model, shock wave model, and granite failure model, as illustrated in Figure 2. 

## 3. Analytical Approach

### 3.1. Equivalent Circuit of High-Voltage Pulse Rock Breaking

**Figure 3:**
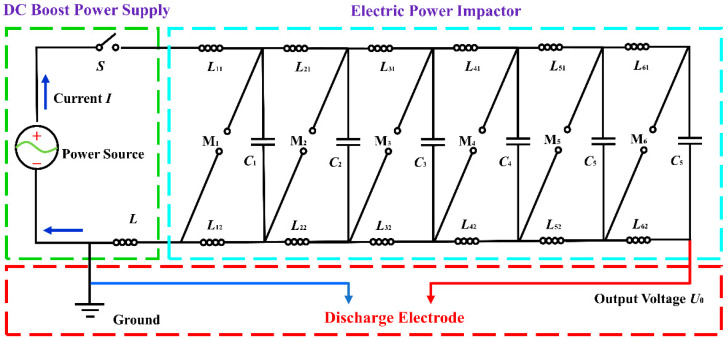
Equivalent circuit of high-voltage pulse instrument: *S*—high-voltage pulse switch; *L*, *L*_11_, *L*_21_, *L*_31_, *L*_41_, *L*_51_, *L*_61_, *L*_12_, *L*_22_, *L*_32_, *L*_42_, *L*_52_, *L*_62_—inductance; M_1_, M_2_, M_3_, M_4_, M_5_, M_6_—spark gap; *C*_1_, *C*_2_, *C*_3_, *C*_4_, *C*_5_, *C*_6_—capacitor.

The equivalent circuit for a high-voltage electrical pulse instrument mainly consists of a DC boost power supply source, an electric power impactor and an output terminal, which is shown in Figure 3. The DC boost power supply in the green area is used to charge the blue area on the right. The capacitors in the blue area store electrical energy for pulse discharge. The electric power impactor (Marx generator), of the capacitive energy type, is often used in high-voltage EPB (Burkin et al., 2009a; Burkin et al., 2009b) [4,18]. Spark gaps are used as voltage protection devices. Inductors are used to adjust the pulse waveform during discharge. The discharge electrodes in the red area are used to release electrical energy to break up the rock.

**Figure 4:**
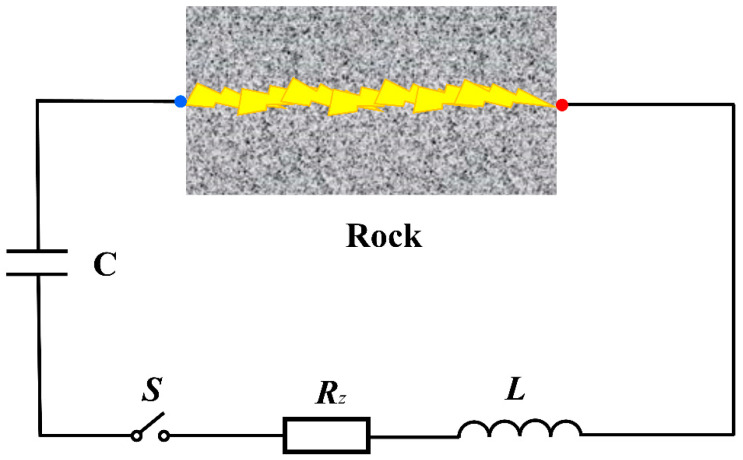
Simplified RLC circuit for electric pulse rock: *S*—high-voltage pulse switch; *R*_z_—resistance of the discharge device; *C*—capacitor; *L*—inductive inductance; Rock—the rock under electropulse.

By a simplification of the discharge device, the electrical pulse rock breaking can be expressed as an RLC discharge circuit (Li et al., 2021a) [23], represented by Figure 4. When the switch is closed, the pulsed current forms a discharge channel in the rock and the rock is considered as a resistance *R*_ch_. It should be noted that when the switch is closed, current *I* would not be generated immediately and the energy of the capacitor only starts to be released once the rock has been electrically breakdown (Zhu et al., 2021b) [20].

This RLC discharge circuit can be represented by Kirchhoff’s equations

(1a) $$L\cdot \frac{di}{dt}+(R_{z}+R_{\mathrm{ch}})\cdot i=U$$

(1b) $$\frac{dU}{dt}=-\frac{i}{C}$$

where *i* is the current in the loop and *U* is the instantaneous voltage of the capacitance; *R_z_* and *R*_ch_ represent the resistance of the discharge device and the arc channel in the rock, respectively. The inductive inductance *L*, the circuit resistance *R_z_* and the capacitance *C* are all intrinsic parameters and do not change with time. The breakdown channel resistance *R*_ch_ adopts a Weizel–Rompe model of impedance (Burkin et al., 2009a; Li et al., 2021a; Xiong et al., 2018) [4,23,32], widely used for plasma channel, which can be expressed in the form of a current integral:

(2) $$R_{\mathrm{ch}}=\frac{K_{\mathrm{ch}}\cdot l_{\mathrm{ch}}}{{\left(\int_{0}^{t}i^{2}dt\right)}^{1/2}}$$

where *K*_ch_ is a spark constant; *l*_ch_ is the length of arc channel.

Combining (1) and (2), the current *i* can be expressed as:

(3) $$\frac{d^{2}i}{dt^{2}}+(\frac{R_{z}}{L}+\frac{K_{\mathrm{ch}}\cdot l_{\mathrm{ch}}}{L{\left(\int_{0}^{t}i^{2}dt\right)}^{1/2}})\frac{di}{dt}-\frac{K_{\mathrm{ch}}\cdot l_{\mathrm{ch}}}{2L{\left(\int_{0}^{t}i^{2}dt\right)}^{3/2}}i^{3}+\frac{1}{LC}i=0$$

The initial value condition for (3) are as follow:

(4) $$\left\{ \begin{array}{c} {i|}_{t=0}=0\\ {\frac{di}{dt}|}_{t=0}=\frac{U_{0}}{L} \end{array}\right.$$

where *U*_0_ is the charge voltage.

The equivalent circuit equation for pulsed rock breaking can be expressed by (3) and the initial value condition (4). This equation can be solved via Matlab.

### 3.2. Mechanical Analysis Model of Electric Pulse

In the previous section, the voltage *U* and the current *i* of the circuit were obtained throughout the discharge process. On this basis, this section allows the volume change *V* and shock wave pressure *P* of the electric channel in the rock to be obtained by energy conservation, EOS (equation of state) for solid media, and the Rankine–Hugoniot conditions (Burkin et al., 2009a; Kratel, 1996) [4,33].

**Figure 5:**
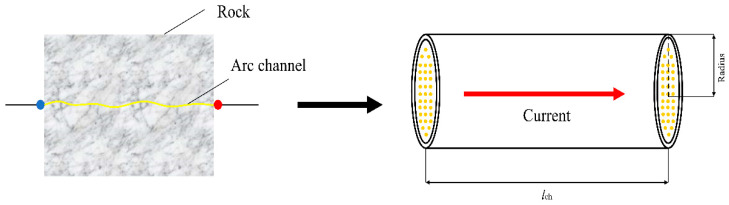
The cylindrical plasma arc channel model in the rock.

When the pulse discharge device starts discharging and creating a current in the circuit, a discharge channel is formed in the rock, as shown in Figure 5. According to the energy balance principle, the energy of the pulsed power supply is injected into the plasma channel, and subsequently the energy of the plasma channel is converted into the internal energy of the plasma channel and the energy of the shock wave during the expansion of the plasma channel.

The total energy of the arc channel *W*_ch_ can be expressed by Ohm’s law

(5) $$W_{\mathrm{ch}}=\int_{0}^{t}i^{2}R_{\mathrm{ch}}dt$$

The channel energy balance equation for *W*_ch_, internal energy *W*_in_ and shock wave energy *W*_pl_ is a key equation to connect plasma channel energy and the discharge energy convert into plasma channel internal energy and mechanical work. The relationship between *W*_ch_, *W*_ws_ and *W*_in_ can usually be described by Burkin et al. (2009a) [4]. An energy conservation equation can be obtained as follows:

(6) $$\frac{dW_{\mathrm{ch}}}{dt}=\frac{dW_{\mathrm{ws}}}{dt}+\frac{dW_{\mathrm{in}}}{dt}$$

The first item d*W*_ws_ = d(*P*·*V*)/(*γ* − 1) describes the change in the channel work performed by the expanding channel at the change of its volume *V* = π*r*^2^*l*_ch_ under the pressure within the channel. *γ* is the effective ratio of specific heats. Here *r* is the channel radius. The second item d*W*_in_ = *P*·d*V* is the plasma channel internal energy approximation. *P* and *V* are the shock wave pressure and volume of the channel respectively. The shock wave pressure, *P*, produced by the plasma channel expansion can be expressed as (Li et al., 2021b) [34]:

(7) $$P=\frac{\gamma -1}{\gamma \times V}W_{\mathrm{ch}}$$

**Figure 6:**
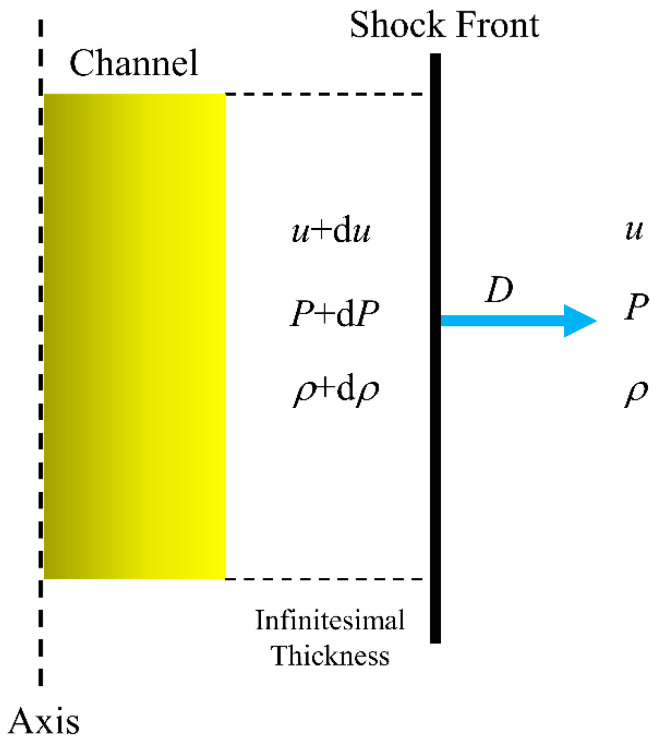
The 2D axisymmetric plasma channel expansion model.

On both sides of the wave front, the shock wave velocity, shock pressure, and medium density are discontinuous, rendering the differential equation meaningless. As a result, consideration must be given to the Rankine–Hugoniot criteria, which are frequently employed to explain the shock wave expansion (Kratel, 1996) [33]. Between the wave front and the arc channel, an infinitesimal thickness is considered, splitting the wave front’s two sides into a disturbed and an undisturbed zone, as illustrated in Figure 6. The disturbance zone experiences an increase in wave velocity, density, and shock wave pressure. The wave front velocity of the shock wave *D* is considered to be the arc burst velocity, where *D* ≫ *u*. The conservation of mass and momentum at the wave front are given the following equations:

(8a) ρ(D−u)=(ρ+dρ)[D−(u+du)]

(8b) (P+dP)−P=ρ(D+u)[(u+du)−u]

The mechanical properties of the rock are defined via the Murnaghan equation, a kind of equation of state, widely used to describe Rock-like media under shock loading (Tang et al., 2008a) [35].

(9) $$P=\psi \left[{\left(\rho_{m}/\rho \right)}^{n}-1\right]$$

where *ρ_m_* is the initial density of the media. *ψ* and *n* are determined via experiment, *ψ* = *ρ*_0_*c*^2^_0_/*n*.

Combining the Rankine–Hugoniot conditions and the Murnaghan equation, the pressure *P* and the arc channel volume *V* are obtained as follows:

(10) $$\frac{dV}{dt}={\left(\frac{n\pi lV}{\rho }\right)}^{\frac{1}{2}}\psi^{\frac{1}{2n}}[{\left(P+\psi \right)}^{\frac{n-1}{2n}}-\psi^{\frac{n-1}{2n}}]$$

The conservation equation of the mass which can be expressed by the volume and shock wave pressure as well is obtained by associating the equation of state and the Rankine–Hugoniot conditions.

### 3.3. Mechanical Damage Process Analysis of Rock by Electric Pulse

This study makes the assumption that there is no medium between the discharge arc and the rock under macroscopic conditions, and that the two are directly coupled. Following the electric explosion in the arc channel, a huge volume of pressurised media expands outward, followed by the application of a strong shock load to the rock. The shock wave disturbs the rock medium, causing partial compression or tension and resulting in the formation of plastic and elastic waves in the rock. The rock material has a reasonably high stiffness (10^10^~10^11^ GPa), and the vibration velocities of the particles on both sides of the wavefront are assumed to be approximate. As a result, reflected waves caused by impedance are omitted from this paper. Arc blast shock waves are regarded to be entirely projected into the rock. In rock media, the relationship between the pressure and propagation velocity of shock waves decaying with distance is expressed as (Wei., 2021; Dai., 2001) [25,26]

(11a) $$\sigma_{r}=P_{d}{\left(\frac{r}{r_{b}}\right)}^{-\alpha }$$

(11b) $$\sigma_{\theta }=bP_{d}{\left(\frac{r}{r_{b}}\right)}^{-\alpha }$$

(11c) $$v_{r}=v_{r0}{\left(\frac{r}{r_{b}}\right)}^{-\alpha }$$

where *P_d_* and *v_r_*_0_ are, respectively, the peak pressure and velocity of the arc burst bombardment projected into the rock; *r* is the distance between the mass point and the discharge centre; *r_b_* is the radius of the arc channel when the peak pressure is transmitted into the rock medium; *σ_r_*, *σ_θ_* and *v_r_* are the radial stress, circumferential stress and mass velocity at distance *r* from the centre of the arc channel in the column coordinates; *b* is the lateral pressure coefficient; *α* is the attenuation index of the shock wave or stress wave.

**Figure 7:**
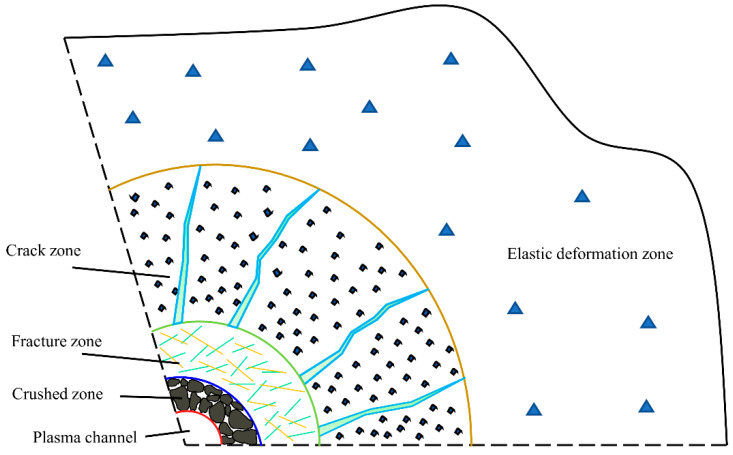
Schematic diagram of damage partitioning of rock electrical pulses.

As illustrated in Figure 7, the disturbed region of an arc explosion can be split into the crushed zone, fracture zone, crack zone, and elastic deformation zone. The crushed zone is located outside the arc and is severely disrupted by the pressure. Under the influence of a high-intensity shock wave, the medium surrounding electric plasma exhibits rheological characteristics, and hence rock medium can be viewed as fluid. The electric blasting action is in a plane strain condition for pulsed current-induced arc columnar expansion blasting in rock media. Given that the material is subjected to a mixture of compression, shear, and tension stresses, the Mises criterion for the plane stress state in the column coordinate system can be used to calculate the damage zone (Dai, 2001; Zhang, 1990) [26,36]. When the high-pressure shock wave in the restricted medium works directly on the rock around the electric channel, it crushes it. Additionally, the area disturbed by the arc shock wave’s pressure is referred to as the high strain rate region (Wei et al., 2021; Li et al., 2017) [25,37]. On this basis, the radius of compacting or crushed zone is calculated as

(12) $$\frac{r_{c}}{r_{b}}={\left[\frac{0.63f_{c}}{BP_{d}}\right]}^{\frac{25}{4-21\alpha_{1}}}{\left[\frac{\alpha_{1}v_{r0}}{r_{b}}\right]}^{\frac{4}{4-21\alpha_{1}}}$$

where *r_c_* is the radius of crushed zone; *f_c_* is the uniaxial compressive strength of rock; *B* is the Mises nominal stress conversion factor, where *B* = 2^−0.5^ × [(1 + *b*)^2^ − (1 + *b*^2^) − 2*μ*/(1 − *μ*)(1 − *b*)^2^]^1/2^; *α*_1_ = 2 + *μ*/(1 − *μ*) is the attenuation index of the shock wave in the crushed zone; *b* = *μ*/(1 − *μ*) and *μ* are the lateral pressure coefficient and Poisson’s ratio.

Some experimental studies have found that pulsed currents striking rocks produce little apparent crushed damage in surrounding rock (Cho et al., 2016; Zhu et al., 2021b) [19,20]. Even when cloud–ground lightning strikes the rock with peak currents of tens of kiloamperes, field observations reveal only approximately 4~10 mm of impact lamellae on the quartz surface (Chen et al., 2017; Elmi et al., 2017) [38,39]. To further investigate the relationship between the shock wave pressure formed by the discharge and the crushed zone, if *r_c_* = *r_b_*, the minimum impact stress *P_c_* to form the crushed zone can be expressed as

(13) $$P_{c}=\frac{0.63f_{c}{\left(\frac{\alpha_{1}v_{r0}}{r_{b}}\right)}^{\frac{4}{21}}}{B}$$

If taking the *f_c_* = 240 MPa and *μ* = 0.2, and if *r_b_* = 3 mm is the radius of the channel at the peak pressure *P_d_*, then the minimum impact stress to form the crushed zone is *P_c_* ≈ 3.3 GPa, which is much higher than the impact pressure generated by the electric pulse device in surrounding rock (Grieve et al., 1996) [40]. This theoretically explains why the electrical pulses cannot produce significant crushed zone in the rock. On the one hand, the shock wave formed by the discharge pulse generates a strong disturbance in surrounding rock, which makes the rock subject to a sharp increase in compressive strength by the effect of strain rate. On the other hand, the radius of the borehole formed by the pulse discharge is small, making the shock wave pressure required to break the rock relatively large.

The shock wave outside the crushed zone attenuates to stress wave which continues to impose tensile damage on the surrounding rock. The area rock subjected to tensile damage belongs to the low strain rate region, thus the effect of strain rate on material strength is ignored. For *P_d_* << *P_c_*, the radius of resulting fracture zone of arc channel hole surrounding rock can be expressed by Dai (2001) [26]

(14) $$r_{t}={\left(\frac{f_{t}}{f_{c}}\right)}^{-\frac{1}{\alpha_{2}}}r_{b}$$

where the *r_t_* is the radius of tensile damage and the *f_c_* is the uniaxial tensile strength of rock. The *α*_2_ = 2 − *μ*/(1 − *μ*) is the attenuation index of the stress wave in the fracture zone. It can be seen from (14) that the range of tensile damage region is not only affected by the tensile strength of the rock, but also related to the compressive strength. This means that the greater the ratio of the rock’s compressive tensile strength, the more significant the tensile damage. This has been verified by some experiments on rocks broken by electric pulse (Cho et al., 2016; Zhu et al., 2021b) [19,20].

Further, the thickness of fracture zone in the electric channel surrounding rock can be calculated as

(15) $$D_{t}=\left[{\left(\frac{f_{t}}{f_{c}}\right)}^{-\frac{1}{\alpha_{2}}}-1\right]r_{b}$$

where *D_t_* is the thickness of the fracture zone in the electric channel surrounding rock.

**Figure 8:**
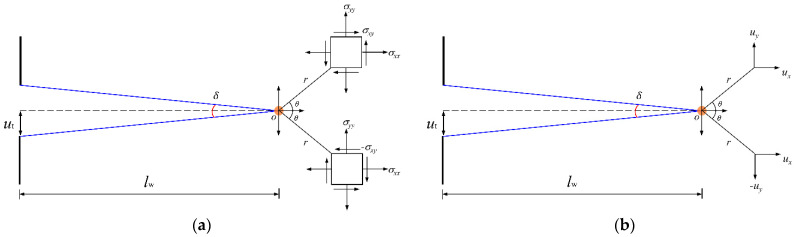
(**a**) The stress field and (**b**) the displacement field with respect to the crack tip.

A sequence of cracks in the surrounding rock form as a result of the shock wave and stress wave. The ensuing expansion of the arc explosion products in the borehole will force the surrounding pressured media extrusion into the surrounding rock fracture area, hence promoting fracture expansion. Figure 8 illustrates the process of crack development, the stress field, and displacement field with respect to the crack tip by selecting one of the cracks and analysing the stress field and displacement field with respect to the crack tip. Given the fracture mechanics description for crack development, various loading configurations result in two distinct modes of crack tip surface displacement (in-plane type of cracks): tensional mode (type I of cracks) and edge-sliding mode (type II of cracks) (Rinne et al., 2020) [41]. If the cracks surrounding the electric channel hole can grow stably under the homogeneous pressure of the confined medium, the edge-sliding mode subjected to in-plane shear stress can be ignored. Thus, the tangential stress at the tip, considering the Type I cracks around the borehole, is often expressed in terms of polar coordinates in 2D (Rinne et al., 2020) [41]:

(16) $$\sigma_{\theta }=\frac{K_{Ι}}{\sqrt{2\pi r}}\cos^{3}\frac{\theta }{2}$$

where *σ_θ_* is the tangential stress of the composite crack; *θ* is the polar angle of the crack tip; *K*_I_ is the dynamic stress intensity factor for type I cracks.

According to (16), it can be found that there exists an optimum angle of crack generation in surrounding rock when the circumferential stress of the surrounding rock is at its maximum. Based on the description of the displacement field at the crack tip, the half of the bottom width of a crack shown in Figure 8 can be expressed as

(17) $$u_{t}=\frac{K_{Ι}}{2G}\sqrt{\frac{l_{w}}{2\pi \cos\frac{\delta }{2}}}\cos\frac{\delta }{4}(\lambda +\cos\frac{\delta }{2})$$

where *l_w_* is the tip angle of cracks; *λ* is the thermal insulation factor of the explosive; and *G* is the shear modulus, where the *G* = *E*/2(1 + *μ*) and *λ* = 3 − 4*μ*.

For a single crack, the pressure at the crack tip will gradually decrease as the crack length develops. Assuming that the ultimate development length of crack is *l_w_*, the corresponding ultimate pressure when the crack stops developing is pw. Considering the isentropic expansion of high-pressure explosion products, and according to the principle of acoustic approximation, the relationship between the ultimate pressure and the peak pressure can be obtained (Yang et al., 2015) [42]:

(18) $$\frac{P_{d}}{P_{w}}={\left[1+\frac{nu_{t}(2D_{t}+l_{w})}{\pi r_{c}{}^{2}}\right]}^{k}$$

where *P_w_* is the ultimate pressure when the crack stops developing; *l_w_* is the length of crack when it stops developing; *n* is the number of major cracks surrounding the borehole; and *k* is the thermal insulation index of the explosion.

Provided that the crack initiation is expressed for type I in terms of the stress intensity factor, then crack initiation occurs when the stress intensity factor reaches its critical value, otherwise called the type I plane strain crack toughness, *K*_IC_. Thus, the condition of crack generation in surrounding rock is given as (Rinne et al., 2020) [41]:

(19) $$K_{I}=K_{\mathrm{IC}}$$

Assuming that the surface angle of the crack tip of surrounding rock in a distant zone is small, meanwhile pw is governed by the residual tensile strength *f_tr_* = *ηf_t_*, with *η* being the residual strength ratio. Given the maximum circumferential stress criterion of crack generation of surrounding rock, if the shocked rock meets the condition of crack generation, the length of the crack can be calculated by correlating (11), (16)–(19):

(20) $$l_{w}=\sqrt{D_{t}{}^{2}+\frac{2G\pi b\left[{\left(\frac{P_{d}}{f_{tr}}\right)}^{\frac{1}{k}}-1\right]r_{c}^{2}}{n(\lambda +1)f_{tr}}}-D_{t}$$

## 4. Rock Electropulse Broken Process

The proposed analytical method is utilised in this study to calculate the final failure rate of a rock caused by a pulse current for a variety of discharge devices and rock mechanics parameters. To advance our understanding of the rock failure process, this article develops a numerical model that simulates the crack formation process when subjected to an electric pulse. It is worth noting that this is necessary in order to obtain more precise simulation results. The completely coupled calculation is used to simulate the RLC circuit, shock wave pressure, and rock failure process.

### 4.1. RLC Circuit and Shock Wave Pressure in the Plasma

**Figure 9:**
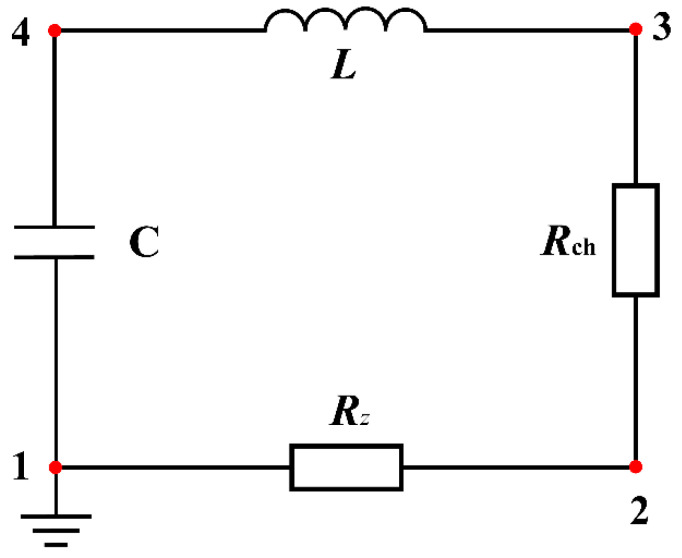
The RLC equivalent circuit for electric pulse in numerical simulation: *R_z_*—resistance of the discharge device; *C*—capacitor; *L*—inductive inductance; *R*_ch_—the equivalent resistance of the rock under electropulse.

The equivalent circuit for electric pulse can be regarded as an RLC loop (Figure 9), which consists of a capacitance *C*, an inductance *L*, a circuit resistance *R_z_* for the electric device and another resistance *R*_ch_ as the arc channel resistance in the rock. The loop in the simulation meets Kirchhoff’s conservation laws and is used to model currents and voltages in the circuit interface. When obtaining the current and voltage in the arc channel, by using Ohm’s laws, the energy within the arc channel can be obtained. Meanwhile, in the same time step, combining the equations of conservation of mass, momentum and energy of the arc channel means that the shock wave pressure formed can be calculated.

### 4.2. Deformation and Damage Evolution of Granite

The mechanical equilibrium of a rock is governed by:

(21) $$\nabla \cdot \sigma +F_{v}=\rho \frac{\partial^{2}u}{\partial^{2}t}$$

where ***σ*** is the stress and *F_v_* is the body force. The mechanical behaviour of rock microscopic damage is essentially deterioration of strength and reduction of bearing capacity. Therefore, the stress–strain relation of rock material obeys the linear elasticity law incorporating a simple class of isotropic damage model:

(22) σ=(1−ω)D:ε

where *ω* is the damage variable; **D** is the elastic stiffness matrix, and **ε** is the strain. It is denoted that compressive stress and strain are positive, and tensile stress and strain are negative. The damage variable *ω* ranges between 0 and 1 (0 < *ω* < 1). When *ω* is 0, the unit is undamaged and when *ω* is close to 1 indicating that the unit is failure. An isotropic damage model is used to simulate the rock failure processes, which are governed by the loading–unloading conditions as (Jirásek and Bauer, 2012) [43]

(23) $$f(\tilde{\varepsilon },\kappa )\leq 0,\frac{\partial \kappa }{\partial t}\geq 0,\frac{\partial \kappa }{\partial t}f(\tilde{\varepsilon },\kappa )=0$$

where $\tilde{\varepsilon }$ is the equivalent strain; and *κ* is an internal variable recording the maximum level of the equivalent strain. with the damage loading function defined as (Jirásek and Bauer, 2012) [43]

(24) $$f(\tilde{\varepsilon },\kappa )=\tilde{\varepsilon }(\varepsilon )-\kappa$$

**Figure 10:**
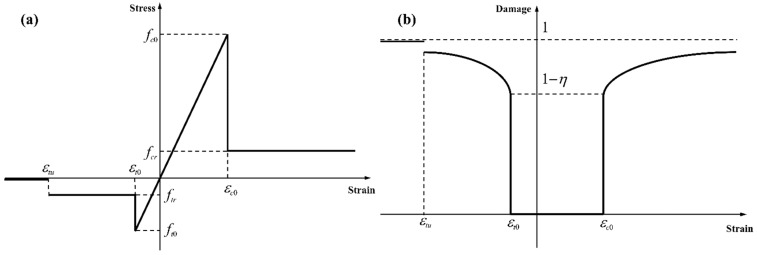
Mechanical response of rock under electric pulse: (**a**) Stress–strain and (**b**) damage–strain relationships in the elasto-brittle damage model of rock materials.

The equivalent tensile and compressive strains, respectively, are defined (Jirásek and Bauer 2012) [43]:

(25a) $$\tilde{\varepsilon_{t}}=-\frac{\left\|\langle -D:\varepsilon \rangle \right\|}{E}$$

(25b) $$\tilde{\varepsilon_{c}}=\frac{\left\|\langle D:\varepsilon \rangle \right\|}{E}$$

where ǁ is the norm operator and 〈 〉 is the Macaulay bracket. The constitutive relationship of a microscopic element under uniaxial tension and uniaxial compression are derived by assuming an elasto-brittle constitutive behaviour shown in Figure 10, such that (Tang et al., 2008b) [44]:

(26a) $$\omega_{c}=\left\{ \begin{array}{c} 0\;\kappa_{c}\leq \varepsilon_{c0}\;\\ 1-\frac{f_{cr}}{E\kappa_{c}}\;\kappa_{c}\geq \varepsilon_{c0} \end{array}\right.$$

(26b) $$\omega_{t}=\left\{ \begin{array}{l} 0\;\kappa_{t}\leq \varepsilon_{t0}\;\\ 1-\frac{f_{tr}}{E\kappa_{t}}\;\varepsilon_{t0}\leq \kappa_{t}\leq \varepsilon_{tu}\\ 1\;\kappa_{t}\geq \varepsilon_{tu} \end{array}\right.$$

where *ω_c_* and *ω_t_*, respectively, are the compressive and tensile damage variables. *ε_c_*_0_ = −*f_c_*/*E* and *ε_t_*_0_ = −*f_t_*/*E*, respectively, are the threshold compressive strain and the threshold tensile strain; *f_c_* and *f_t_* are the tensile and compressive strengths, respectively; *f_cr_* = *ηf_c_* and *f_tr_* = *ηf_c_* are the residual tensile and compressive strengths, respectively, where *η* is the residual strength ratio; *κ_c_* and *κ_t_* are the internal variable for tensile and compressive conditions, respectively.

### 4.3. Model Setup

**Figure 11:**
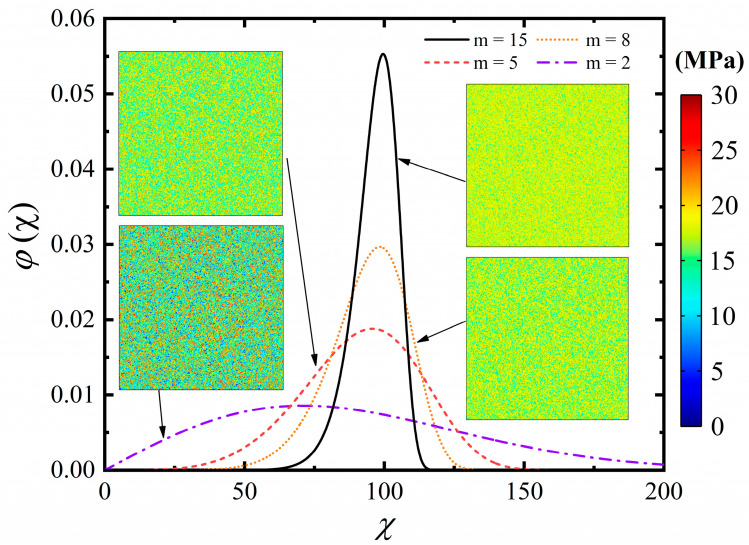
The number of elements with a particular value as a function of that value for different homogeneity indices (m = 2, 5, 8, and 15) at a given scale parameter of *β* = 100. Snapshots of the numerical specimens are also provided to show the distribution of tensile strength within specimens characterized by different homogeneity indices (m = 2, 5, 8 and 15).

Rock is a heterogeneous material, and it is the heterogeneity that causes the rock to fracture and crack via the formation, extension and coalescence of microcracks in the rock. This means that, in the absence of heterogeneity, there will be no damaged localization and the local behaviour of the homogenous model is replicated at the macroscopic scale. To reflect the rock’s heterogeneity at the mesoscale, the spatial distribution of mechanical characteristics is assigned based on the 2-parameter Weibull function with threshold values (Weibull, 1951) [45] as

(27) $$\varphi (\chi )=\frac{m}{\beta }{\left(\frac{\chi }{\beta }\right)}^{m-1}\exp\left(-{\left(\frac{\chi }{\beta }\right)}^{m}\right)$$

where *χ* is the scale parameter of an individual element; *β* is the scale parameter giving the characteristic value of distribution *χ*, which is equal to 100 in this paper, and m is the homogeneity index describing the spatial concentration. The inhomogeneous tensile strength of rock is shown in Figure 11, in which it can be found that with the increase of the scale parameter *β*, the tensile strength property of the rock will be more similar to the mean value. Based on the definition of the Weibull function, the value of homogeneity index m should be greater than 1.

**Figure 12:**
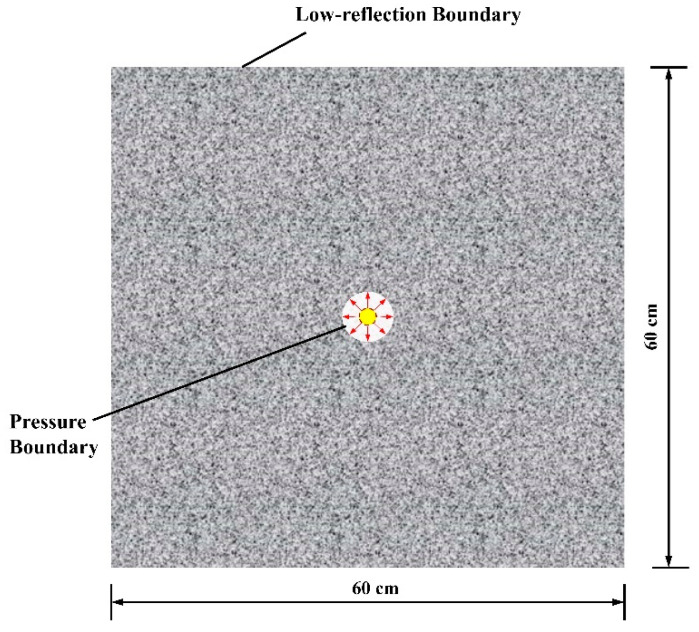
Mechanical equivalent model of electric pulse rock.

As illustrated in Figure 12, this model is treated hypothetically as a two-dimensional square rock domain (horizontally placed) in granitic rock and a borehole. The out-of-plane thickness is assumed to be equal to the plasma channel’s length, *l* = 0.08 m. The spatial domain’s distribution of compressive and tensile strengths conforms to the Weibull function with m = 5. A surface traction force model is utilised to mimic the rock collapse process caused by high-voltage pulse fragmentation. To eliminate interference from reflected waves and to simulate the real-world circumstances of an electric pulse rock experiment, the model’s boundary was regarded as a low-reflection boundary. The model’s length and borehole radius are 600 mm and 3 mm, respectively. The shock wave pressure calculated within the arc channel is utilised to determine the borehole’s boundary load.

To improve convergence, the model domain is spatially discretized using an unstructured grid and a mesh refinement around the borehole. The time–domain computation is performed in an adaptive step fashion with a preset maximum time step of *t*_max_ = 0.1 µs and a tolerance of 0.01, while the backwards differentiation formula is used to calculate the time-stepping approach, ensuring numerical convergence and stability.

The numerical simulation takes place in a single stage. Each calculation is fully coupled. That is, in a single time step, the voltage and current in the circuit are computed simultaneously with the volume of the arc channel and shock wave pressure, as well as the mechanical characteristics of the granitic rock. The discharge device, arc channel, and granite specifications are presented in Table 1, which were chosen from prior literature to be within a suitable range (see Table 1).

## 5. Results and Discussion

### 5.1. Verification

**Figure 13:**
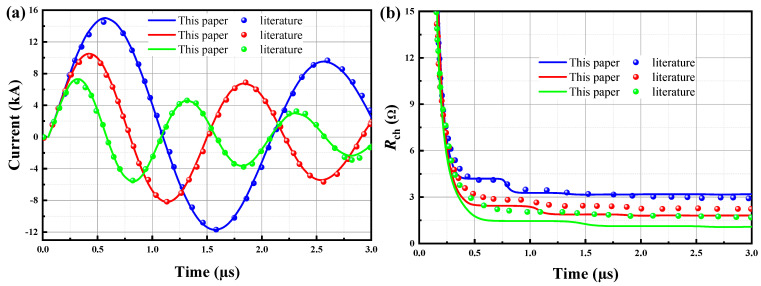
Validation of this paper’s results against the simulation results from Literature [4] for the electric pulse loop. (**a**) Wave form of current I and (**b**) channel resistance *R*_ch_, where *U*_0_ = 280 kV, *L* = 5 µH, *l* = 2.5 cm, *C* (nF): blue line—5, red line—10, green line—20.

By using a numerical method, Burkin et al. (2009a) [4] predicted the power characteristics of electro burst in solids. In this paper, the proposed model is calculated by using the same discharge parameters as those of Burkin et al. (2009a) [4]. Figure 13a shows the current waveforms of discharge channel is approximately the same. It can be found that under the condition of only increasing the capacitance *C*, the peak current *I_m_* and wave period in the plasma channel subsequently increase. The plasma channel resistance *R*_ch_ is depicted in Figure 13b. As shown in Figure 13b, it can be found that the channel resistance shows slight differences between this paper and Burkin et al. (2009a) [4] a result of the difference of selection of the initial value of electric resistance *R*_ch_. Figure 13b shows that the resistance has a steep drop in the first 0.5 µs, and then changes very little, where the granite can be considered as breakdown. Interestingly, the larger the capacitance value C, the higher the breakdown resistance.

In recent decades, a new method called pulse discharge technology (PDT), in which the conversion of stationary electric energy stored in a capacitor into an arc or corona electric discharge energy is dissipated via electrodes in a few micro-seconds, has been applied to crush rocks and some brittle materials (Bakholdin and Dzhantimirov, 1998) [48].

**Figure 14:**
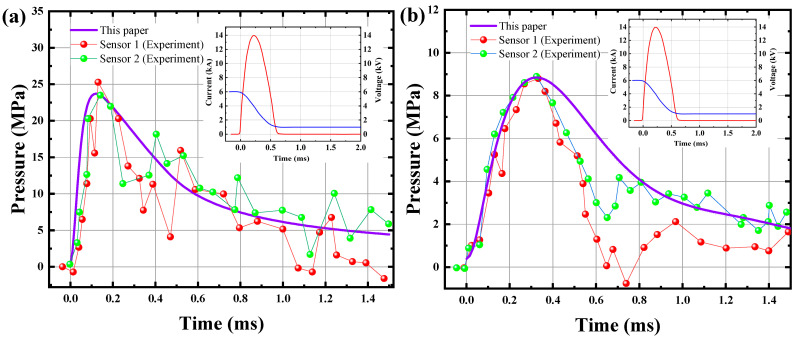
Validation of this paper’s results against the experiment results from Park et al. (2011) [49] for shock wave pressure. (**a**) Chamber diameter: 110 mm (**b**) Chamber diameter: 250 mm.

In order to examine the characteristics of shock waves generated by PDT, Park et al. (2011) [49] performed laboratory pulse discharge experiments using PDT equipment. Since the shock waves propagate and attenuate in space, Park et al. (2011) [49] set up two sensors to monitor the time–course variation of the stresses in differently positions. As shown in Figure 14, the results of this paper are compared with the experimental results of Park et al. (2011) [49]. From the figure, it can be found that the results of this paper are in close accordance with those of Park et al. (2011) [49], showing even more consistency at the smaller chamber diameter. This is because the decay rate of the shock wave is not constant in space and time. It can be seen from the figure that the peak pressure received by the sensors is influenced by the chamber diameter, the longer the chamber diameter the smaller the peak pressure and vice-versa.

**Figure 15:**
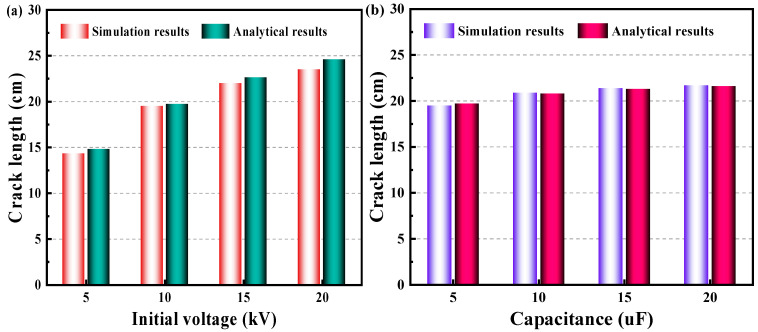
Validation of this paper’s results for crack length under different (**a**) initial voltage and (**b**) capacitance by using simulation approach and analytical approach, respectively.

The purpose of this research is to investigate the damage zone and length of cracks in granite when subjected to an electropulse. This research uses an analytical approach to determine the final length of the cracks and the damage radius. To elucidate the granite failure process further, this study provides a numerical model that incorporates circuit, shock wave, and mechanics. As far as the authors are aware, there is currently no experimental data or analytical model for the damage zone or fracture length in granite when subjected to an electropulse that incorporates circuit, shock wave pressure, and mechanical influence. Thus, the fracture length must be compared using the analytical and simulated methodologies described in this research, the results of which are presented in Figure 15. It is recognised that circumferential cracks rarely develop uniformly, and the dominating cracks emerge prominently within the thick crack group. According to the experimental literature (Li et al., 2021a) [23], there are 4~12 prominent cracks. Table 1 contains the parameters used to validate the model.

The analytical study in this work approximately calculates the final length of cracks under electropulse. The analytical solution and numerical simulation of the crack length were calculated under different initial voltage and capacitance conditions as shown in Figure 15. Figure 15a shows that there is a gradual increase in the length of dominant cracks with the initial voltage improving. The increase in the length of dominant cracks gradually slows down with the increase in initial voltage. Figure 15b reveals that there was not significant change in the length of dominant cracks when the capacitance was boosted, which means that increasing the voltage of the discharge device is more effective than building up its capacitance for electric pulse rock breaking. It can be seen that there is a reasonable degree of consistency between the results of the analytical method and simulation.

### 5.2. Granite Failure Process Analysis

**Figure 16:**
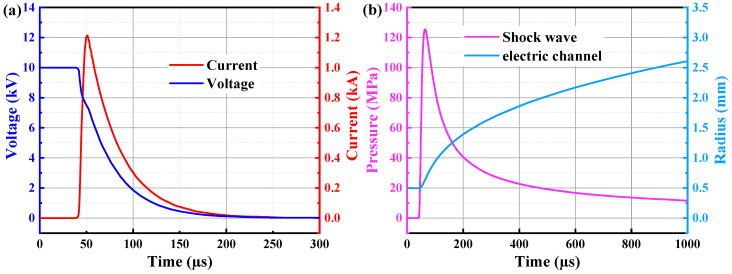
(**a**) Current and Voltage of circuit, (**b**) shock wave pressure and radius of electric channel under electropulse.

The proposed model is deployed in this section to analyse the granite failure process under electropulse, focusing on the development of cracks and displacement. Figure 16a illustrates the current and voltage in a circuit when an electropulse is applied. Prior to approximately 40 µs, the granite had not been broken down and the loop circuit had not been established in such a way that current and voltage remain constant. Between around 40 and 45 µs, a rapid drop in voltage and a sharp spike in current can be observed. The current then continues to rise and stabilises at about 1.2 kA after about 50 µs. After 50 µs, the current and voltage gradually decrease until they reach zero at roughly 250 µs. In Figure 16b, it is shown that the number of shock wave pressures increases rapidly after about 40 µs, reaches a peak at around 70 µs, and then progressively decreases. Simultaneously, after around 40 µs, the radius of the plasma channel, as well as the rate at which the radius grows, begins to diminish. Within 1000 µs, the radius of the plasma channel increases from 0.5 mm to around 2.6 mm. What is notable about this figure is the high correlation between the current and the shock wave, which has also been observed in prior work (Lee et al., 2019) [50].

**Figure 17:**
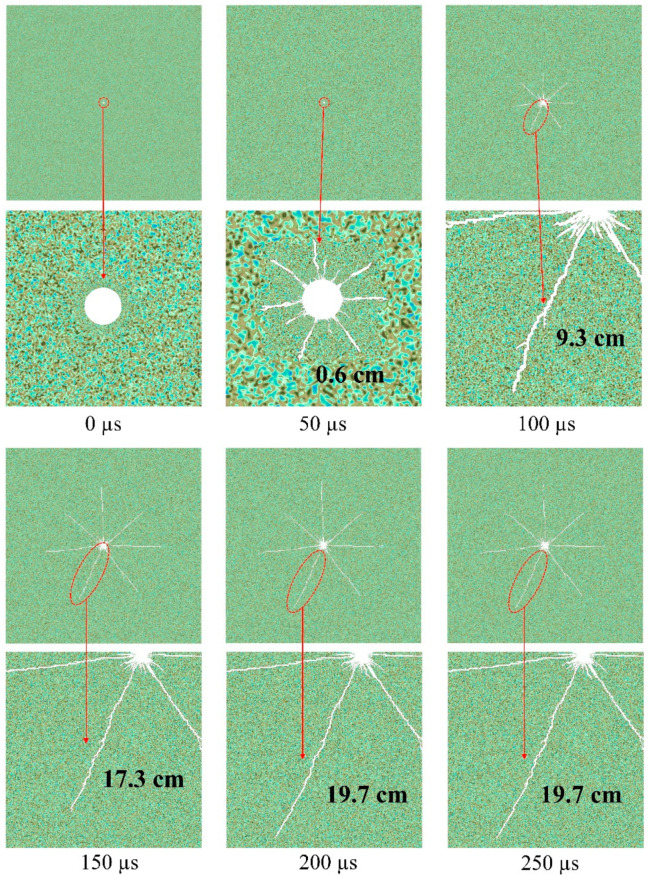
Granite failure process and development of cracks under high-voltage electropulse with time.

**Figure 18:**
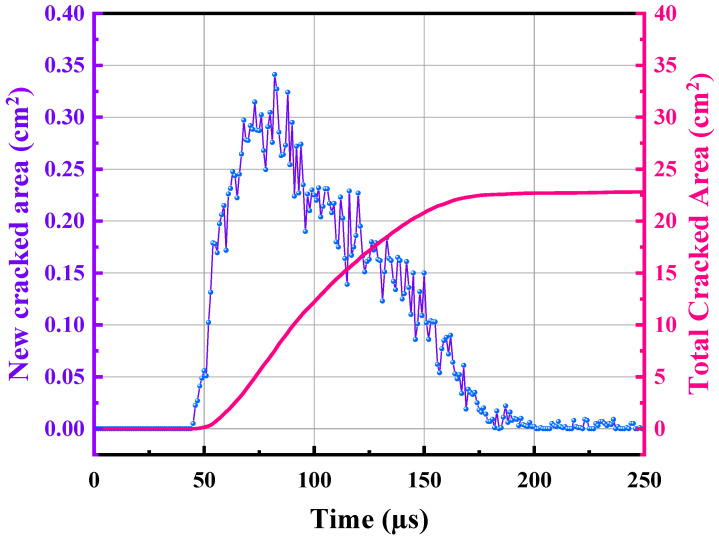
Temporal evolution of new cracked area and total cracked area in the granite under high-voltage electropulse.

Figure 17 illustrates the rock failure process and the formation of cracks over time and space. The shock wave pressure and current reach their peak value at roughly 50 µs, although the average length of the cracks is approximately 0.6 cm. It is apparent that seven dominant cracks developed from the numerous cracks surrounding the borehole. Using one of the major cracks as an example, the dominant cracks extend to roughly 9.3 cm at 100 µs. Subsequently, as can be seen in this figure, this prominent crack continues to expand. At 150 s, this crack has grown to around 17.3 cm in length. At roughly 200 µs, this crack grew to approximately 19.7 cm in length. Following that, growth of the crack’s length halted. The formation of thick cracks around the borehole may clearly be attributed to the direct influence of the shock wave pressure. The simulation revealed that as the shock wave pressure reached its peak, these dense cracks rapidly formed in their entirety. However, the time scale for the growth of dominant cracks is substantially longer than that of dense cracks. Indeed, the prevailing cracks are not totally determined by shock wave pressure, but also by the quasi-static action of the confined medium or expanded products. This quasi-static effect will continue to pressurise the system, causing the cracks to propagate in the direction of the optimal crack. The best orientation of the fracture is determined by its angle and the strength of the granite surrounding the crack tip. As illustrated in the image, secondary fractures can form within dominant fissures, however these secondary cracks are typically brief. To further investigate the development of cracks in the granite, this section monitors the new cracked area and total cracked area at time periods between 0 µs to 250 µs in Figure 18. The figure shows that the cracks start to grow at about 40 µs, and the growth rate reaches a maximum of about 33 m^2^/s at approximately 75 µs. Subsequently, the rate of cracked growth gradually drops and decreases to almost zero at about 200 µs. It can be seen that the total cracked area eventually reached about 22.5 cm^2^ at 200 µs. By the relationship between the total cracked area *S_c_* and the number *φ* and length *l_w_* of dominant cracks, ignoring the dense cracks surrounding the borehole, the width of cracks *d_cr_* can be estimated as

(28) $$d_{cr}=\frac{S_{c}}{l_{w}\varphi }\approx 1.6\;\mathrm{mm}$$

where *d_cr_* is the width of the cracks; *S_c_* is the total cracked area and *φ* is the number of dominant cracks.

**Figure 19:**
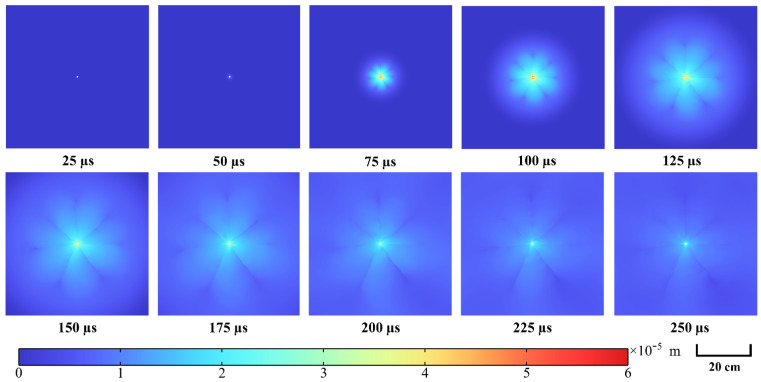
Cloud diagrams of development of granite displacement under high–voltage electropulse at different time.

Figure 19 presents the development of granite displacement under high-voltage electropulse at different time. It can clearly be seen that the disturbance of granite appears semi-circular and is obviously influenced by the propagation of shock waves. Seven main cracks can be distinctly observed. The granite surrounding the borehole is most significantly disturbed and the disturbance decreases with increasing distance of borehole. After about 200 µs, the cloud diagrams show that the displacement of granite tends to steady and the disturbance to the granite from the shock wave almost vanishes. The propagation and diffusion of the shock wave and the process of crack extension in the granite can be clearly observed from the displacement cloud diagrams.

### 5.3. Effect of Heterogeneity of Tensile Strength

**Figure 20:**
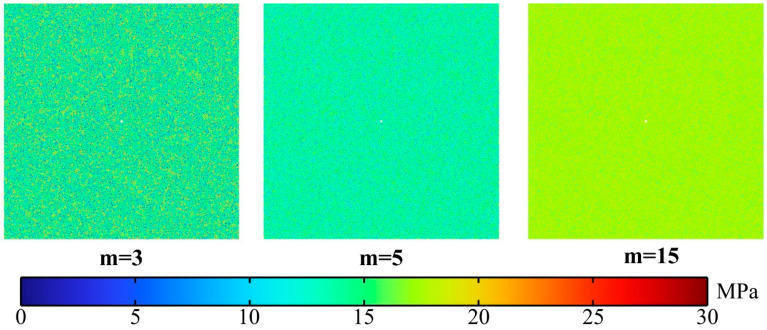
Cloud diagrams of the tensile strength of granite for different homogeneity indices m = 3, 5, 15.

For rock, failure is not only a state, but also a microscopic to macroscopic trans-scale process. Mineral crystals and cements in granite with different mechanical properties and the existence of initial defects lead to great spatial heterogeneity in physical properties of rock. This heterogeneity of rock may affect granite failure processes under high-voltage electropulse. Thus, this section will attempt to assess whether and how the heterogeneity of granite affects the development of cracks. Figure 20 indicates the distribution of tensile strength of granite under different homogeneity indices m = 3, 5, 15. It can be clearly seen from the cloud diagrams that with increasing m, the distribution of tensile strength in the granite are more concentrated and it is worth mentioning that the tensile strength of all three cloud diagrams tend to be 18 MPa.

**Figure 21:**
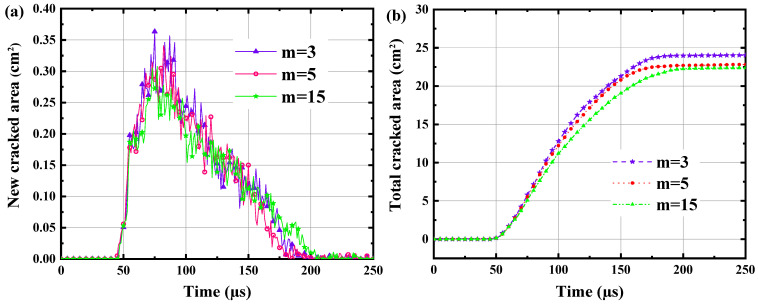
Temporal evolution of (**a**) new cracked area and (**b**) total cracked area in the granite under high voltage electropulse for different homogeneity indices m = 3, 5, 15.

**Figure 22:**
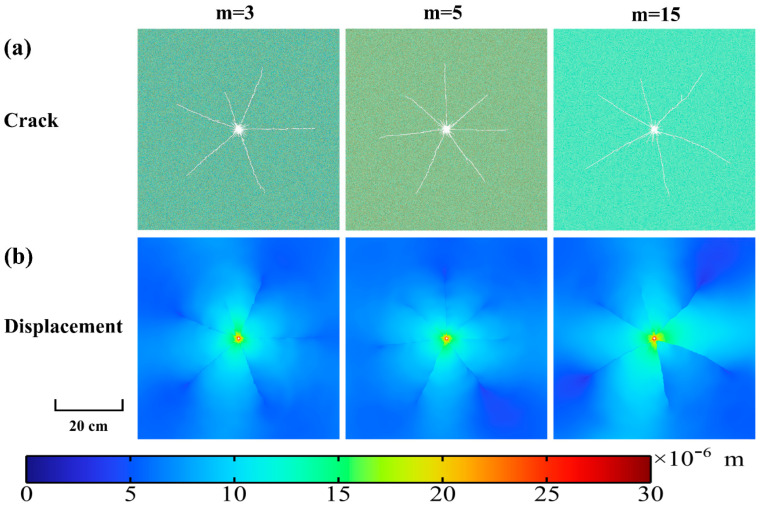
(**a**) Distribution of final cracks and (**b**) cloud diagrams of final displacement in granite under electropulse for different homogeneity indices m = 3, 5, 15.

Figure 21 indicates the temporal evolution of new cracked area and total cracked area in the granite under high-voltage electropulse at different homogeneity indices. It can be noticed from Figure 21a that the inhomogeneity index hardly affects the growth rate of cracks, but the smaller the inhomogeneity index the greater the fluctuation of the development rate of cracks. From Figure 21b, the effect of heterogeneity on the total crack length is minimal. It can be clearly found that the displacement on both sides of the crack is discontinuous. From the figure, it can be found that the cracks start generating at about 40 µs and stop growing at about 200 µs. The peak development rate of new cracked area is about 30 cm^2^ at about 75 µs and the total cracked area reaches a peak of about 22.5 cm^2^ at about 200 µs. Figure 22a shows that greater heterogeneity may result in an uneven distribution of lengths of dominant cracks. For m = 3, it can be seen that the length of one dominant crack is significantly smaller than that of the others. However, what is interesting in Figure 22b is that the total cracked area is scarcely affected by the homogeneity index. This means that once a dominant crack is significantly small the others would be longer. The displacement distribution of granite is almost the same under different heterogeneous conditions, but there are differences in the distribution of the dominant cracks’ development direction and the length of the dominant cracks. In Figure 22, it can be found that the difference in heterogeneity does not affect the number of dominant cracks *φ*. The number of dominant cracks *φ* generated in the granite subjected to high voltage electropulses is approximately 6~7.

### 5.4. Discussion

The analytical study in this work is based on a multi-physics and multi-stage technique for addressing the rock damage process caused by an electropulse, which includes an RLC equivalent circuit, a shock wave model in a plasma channel, and the rock damage evolution. To determine the instantaneous current in the rock’s electric plasma channel, the suggested analytical approach first calculates the equivalent electric circuit of the high-voltage discharge device. The second stage obtains the shock wave pressure induced by the electropulse, as well as the expansion rate and volume of the plasma channel, using the instantaneous current from the previous stage and taking into account the conservation of energy, mass, and momentum in the plasma channel. The shock wave pressure is then transmitted into the rock, causing it to vibrate and disturb the surrounding rock. To further examine the damage process and failure pattern of rock, this paper divides the damage zone generated by shock wave pressure into several regions: crushed zone, fracture zone, crack zone, and elastic deformation zone. Several prior investigations have established, through experimental observation, that electropulses rarely produce crushing damage to rock (Cho et al., 2016; Chen et al., 2017; Elmi et al., 2017) [19,38,39]. This paper also exhibits this phenomenon through the use of an analytical method. Following that, this research examines the radius of the fracture zone caused by an electrical pulse. The fracture zone is a direct result of the shock wave, and its primary characteristic is the dense network of short cracks in the surrounding rock (Kutter and Fairhurst, 1971) [31]. Additionally, this article analyses the final length of prominent cracks when subjected to an electropulse. The dominating cracks are subjected to the function of electric explosive products, which, in the form of explosive gases and high-temperature plasma, indirectly drive the growth of major fractures (Yudin et al., 2019) [9]. These electric explosive compounds are injected into boreholes and cracks, where they continue to develop the primary cracks via quasi-static action. At this moment, the pressure in the crack tip continues to decline as dominant cracks grow. When the tension in the fracture tip is equal to or less than the crack toughness, the development halts, signalling the conclusion of the rock’s failure process under high voltage electropulse.

**Figure 23:**
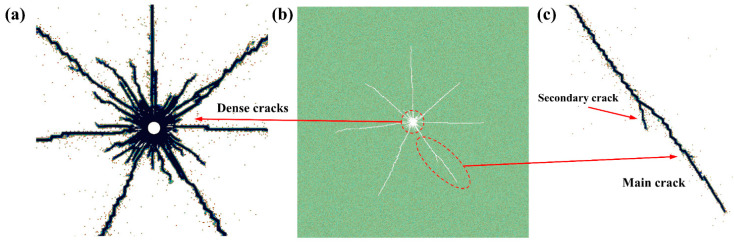
Distribution of cracks in granite under electropulse charge: (**a**) dense cracks near the electric channel hole, (**b**) final distribution of cracks, and (**c**) a main crack and a secondary crack.

However, it should be noted that the proposed analytical approach can only assess the ultimate form of rock failure. The damaging process in the time region of a rock subjected to an electropulse must be simulated numerically. As a result, this article constructs a finite element model to further investigate the mechanism of rock degradation caused by a high voltage electropulse. This research mimics the propagation of cracks and displacement of granite using granite as an example and considering the variety of the rock components in the space. Granite’s damage progression and failure criteria are seen as a type of brittle failure criterion. The proposed model in this study is a fully coupled model that includes an electropulse charge circuit, shock wave pressure, and the spatio-temporal evolution of granite damage. The simulation findings demonstrate a high degree of congruence with the analytical solutions. Figure 23 illustrates the final damage state of granite when subjected to an electric pulse. As seen in Figure 23, the granite contains a dense network of short cracks near the electric channel hole and six longer main cracks. Meanwhile, it is worth noting that certain secondary fractures form adjacent to the primary fissures. The consistency between the numerical and analytical results illustrates the efficacy and accuracy of the mechanism analysis performed in this study for rock subjected to a high voltage electropulse.

**Figure 24:**
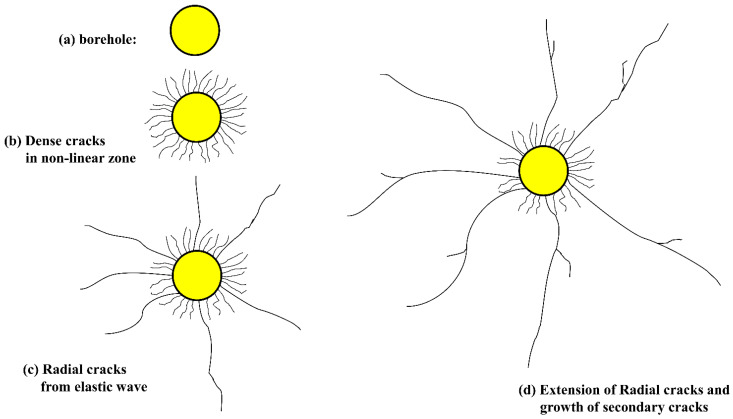
Consecutive stages in the fracture and crack development process of an electropulse in rock: (**a**) electric channel hole, (**b**) growth of dense crack, (**c**) radial dominant cracks development, and (**d**) extension of radial cracks and secondary cracks generated in main cracks.

Notably, raising the voltage of the discharge device, rather than the capacitance, often improves the efficiency of rock breakage under optimum conditions. However, under the condition that the rock is not completely penetrated, it is worth noting that increasing the capacitance of the discharge device, rather than the voltage, is more efficient at increasing efficiency. The rock is supposed to be entirely electrified in this paper until it has a complete breakdown in the final. Additionally, this article summarises the damage evolution of rock when subjected to a high voltage electropulse, as illustrated in Figure 24. At the start of the discharge, the rock is not entirely penetrated, and over time, an electric channel borehole forms (Figure 24a). After the rock has been entirely pierced, a current and shock wave are generated within it. Disturbed by the intense shock wave, a dense network of small cracks forms near the borehole depicted in Figure 24b. Meanwhile, several explosive products such as high-temperature gases, plasma, and spark can significantly improve the efficiency of electric blasting by generating six to seven longer prominent radial cracks in the rock, as illustrated in Figure 24c. On the primary cracks in Figure 24d, some secondary cracks are randomly produced. The effect of these explosive products on the extension of cracks can be thought of as an elastic wave with a quasi-static role. This quasi-static movement is critical for the expansion of cracks in rock. Thus, in order to maximise the energy released by a high-voltage electrical pulse, it is critical to lengthen the discharge duration in order to create more effective explosive products. Additionally, altering the borehole’s pressure-bearing material can increase the release of electric discharge energy. For instance, similar to the drilling–blasting approach, the borehole can be prebored and the pressurised medium can be water, which also improves the length growth of main fractures. It is critical to optimise the device’s electric discharge structure and high-voltage pulse technology in subsequent work based on the process described in this research.

## 6. Conclusions

The primary aim of this analysis was to investigate the mechanism of rock failure under the influence of a high-voltage electropulse using a proposed analytical approach and simulations. The analytical technique suggested here integrates the RLC equivalent circuit, a shock wave model in electro channel plasma, and rock failure characteristics to describe the damage to rock induced by a high-voltage electropulse. To further elucidate the rock failure process, using granite as an example, a numerical model was built to examine the growth process of cracks when subjected to an electric pulse. The following are some of the most significant conclusions gained:

(1) The analytical approach and simulations described here are highly consistent with the prior research. The proposed methodologies are capable of accurately calculating the progressive failure process of granite and revealing the mechanism of rock failure under electropulse, thereby guiding electropulse breaking technology and estimating the degree of rock damage.
(2) Under the conditions described in this study, and using granite as an example, the granite is impacted by a discharge device (Marx generator) with an initial voltage *U*_0_ that is 10 kV and a capacitance *F* that is 5 µF before it begins to degrade at about 40 µs after discharge, with the current reaching its peak at approximately 50 µs. The shock wave pressure then attains a peak at about 70 µs. Dense short cracks form around the granite and the dominant cracks grow to an average length of about 20 cm at around 200 µs. The crack width *d_cr_* is predicted to be approximately 1.6 mm.
(3) Increasing the initial voltage *U*_0_ of the discharge device has a more significant effect on the breaking granite than increasing the capacitance *C*. The extent to which granite is damaged by an electric pulse is determined mostly by the distribution of tensile stress and the tensile strength.
(4) The lack of coherence in the rock’s tensile strength can result in variations in the length of the dominant cracks. However, heterogeneity has a negligible effect on the rate of crack propagation, the total cracked area, or the number of major cracks. There are around six to seven dominant cracks.

## Figures and Tables

**Table 1:** Parameters of electric pulse granite.

	Parameter	Values	Source
Electric circuit	Initial voltage, *U*_0_ (kV)	10	Burkin et al., 2009a [4]; Li et al., 2019 [46]
	Capacitance, *C* (µF)	5	
	Inductance, *L* (uH)	5	
	Circuit resistance, *R_z_* (Ω)	1	
	Spark constant *K*_ch_ (V·s^1/2^·m^−1^)	611	
Arc channel	Initial channel radius, *r*_0_ (mm)	0.5	Burkin et al., 2009a [4]; Li et al., 2019 [46]
	Ratio of specific heat, *γ*	1.1	
	Material bulk constants, *ψ* (GPa)	8.5	
	Material coefficient, *n*	4	
Granite	Young’s modulus, *E* (GPa)	35	Li et al., 2015 [21]; Xu et al., 2018 [47]
	Density, *ρ* (kg/m^3^)	2700	
	Tensile strength, *f_t_* (MPa)	18	
	Compressive strength *f_c_* (MPa)	240	
	Residual strength ratio *η*	0.1	
	Poisson’s ratio, *μ*	0.2	
	Homogeneity index, m	5	

## Data Availability

Data sharing not applicable.
